# Achieving High-Quality Formed Hastelloy X Cladding Layers on Heterological 50CrVA Surface by Optimizing Process Parameters in Directed Energy Deposition

**DOI:** 10.3390/mi16101110

**Published:** 2025-09-29

**Authors:** Liming Xia, Hongqin Lei, Enjie Dong, Tingyu Chang, Linjie Zhao, Mingjun Chen, Junwen Lu, Jian Cheng

**Affiliations:** 1 State Key Laboratory of Robotics and System, Harbin Institute of Technology, Harbin 150001, China; 2 School of Mechatronics Engineering, Harbin Institute of Technology, Harbin 150001, China; 3 Aircraft Repair & Overhaul Plant, Civil Aviation Flight University of China, Guanghan 618307, China

**Keywords:** directed energy deposition, Hastelloy X, 50CrVA, process parameter optimization, performance verification

## Abstract

Hastelloy X exhibits outstanding thermal fatigue resistance, making it a promising material for repairing 50CrVA landing gear via directed energy deposition (DED). However, the substantial differences in composition and thermophysical properties between 50CrVA and Hastelloy X pose challenges by affecting interfacial microstructure and surface quality. This study investigates the effect of DED process parameters (laser power *p*, powder feed rate *f*, scanning speed *v*, and overlap rate) on the dilution ratio (*η*), microscopic morphology, surface flatness (*ζ*), and porosity of Hastelloy X claddings on a 50CrVA substrate. An optimization methodology integrating thermal–flow coupled simulation models and orthogonal experiments is developed to fabricate high-quality claddings. Furthermore, the corrosion–wear performance of the claddings is evaluated. The results indicate that the *η* of a single track increases with higher *p* or lower *f*, while it first increases and then decreases with the increase in *v*. Ablation marks tend to occur at excessive *p* or insufficient *f*, while low *v* causes surface ripples. The *ζ* of a single layer initially improves and subsequently deteriorates with increasing overlap rate. Porosity is significantly influenced by *p* and *f*. The optimal *p*, *f*, *v*, and overlap rate are 1600 W, 2.4 g/min, 240 mm/min, and 55%, respectively. The wear resistance of the cladding is nearly identical to that of the substrate, while corrosion resistance is significantly improved. This work provides a theoretical foundation for high-performance repair of 50CrVA landing gear in aircraft.

## 1. Introduction

Due to the fact that surface defects can lead to severe performance failure of workpieces, and direct replacement of core components would result in a huge waste of resources, the repair of these damaged parts is of vital importance [1,2]. 50CrVA, a high-strength and high-fatigue-resistance material, is widely used in the aerospace and mechanical manufacturing fields, such as in aircraft landing gears. Directed energy deposition (DED) has been extensively applied in component repair within the aerospace, transportation, and related industries [3,4,5,6,7], offering a new approach for restoring aircraft landing gears to thereby reduce economic losses. Compared to traditional additive manufacturing technologies, such as thermal spraying [8], electroplating [9], and arc welding [10], DED offers distinct advantages, including superior interface strength, a reduced heat-affected zone, and precise quality controllability [11]. In landing gear repair, Hastelloy X can be rapidly melted and form a dense, low-dilution interface with the 50CrVA substrate, making it an ideal material for DED. However, the incompatibility arising from differences in properties such as thermal conductivity and coefficient of thermal expansion between the two materials poses challenges for controlling the surface quality of the cladding layer.

The deposition of high-quality Hastelloy claddings on diverse substrate materials has always been a hot topic in the field of DED-based repair. Guo et al. [12] and Zhang et al. [13] investigated the crack formation mechanism in Hastelloy X deposited on the substrates of Hastelloy X and Inconel 718, respectively. Both studies found that cracks preferentially nucleate at high-angle grain boundaries. During DED on QT500-7 cast iron, Chen et al. [14] introduced Al/Ti elements into Hastelloy powder and discovered that the resultant reinforced particles enhanced both the yield strength and ductility of the cladding, while significantly reducing internal porosity. Xia et al. [15] improved the mechanical properties of a Hastelloy X cladding on Q235 steel through heat treatment, resulting in a more homogeneous microstructure. Meanwhile, ultrasonic-vibration-assisted deposition [16,17] significantly enhanced the high-temperature deformation resistance and fatigue life of Hastelloy X claddings deposited on SS304 and 316L substrates. Jinoop et al. [18] obtained a range of process parameters for depositing a single-layer crack-free Hastelloy X on an SS304L substrate. Based on the aforementioned review, it can be concluded that current studies primarily focus on defect formation mechanisms and surface quality optimization of Hastelloy claddings deposited on nickel-based alloys, low-carbon steels, and cast-iron substrates. However, as a critical material for aircraft landing gear components, 50CrVA substrate has not yet attracted research efforts regarding its DED process with Hastelloy X in either domestic or international academia. Therefore, this work investigates the DED mechanisms and process parameter optimization for depositing Hastelloy X claddings on the 50CrVA substrate, aiming to provide a theoretical foundation and parametric support for the repair of aircraft landing gear.

Additionally, obtaining high-quality claddings through process parameter optimization is an effective approach to achieving high-performance additive repair. Gonnabattula et al. [19] optimized process parameters based on the geometric characteristics of the single-track cladding cross-section on Ti6Al4V substrates. Several studies [20,21] employed response surface methodology (RSM) to optimize the dilution ratio and porosity by regulating laser power (*p*), overlap rate, and *Z*-axis offset. Feenstra et al. [22] utilized a dataset of 297 samples and artificial neural networks (ANNs) to investigate the coupled effects of process parameters (e.g., laser power and spot diameter) on cladding morphology. Ghasempour-Mouziraji et al. [23] employed machine learning trained with simulation-derived data to improve the efficiency of process parameter optimization. Chen et al. [24] used orthogonal experimental design to optimize porosity and deposition efficiency in multi-layer cladding processes. Chandra et al. [25] achieved step-by-step parameter optimization from single-track to multi-layer processing. It can be observed that current research on process parameter optimization primarily concentrates on the application of discrete methods, such as RSM and ANNs. These approaches typically target individual process stages, including either single-track or multi-layer cladding. However, these methods require extensive experimental data, resulting in low optimization efficiency, and neglect the influence of heat accumulation between various processing stages. Therefore, it is necessary to propose an optimization method that integrates multiple physics-coupled numerical simulations with orthogonal experiments to rapidly determine the optimal process parameters for multi-layer cladding.

**Figure 1:**
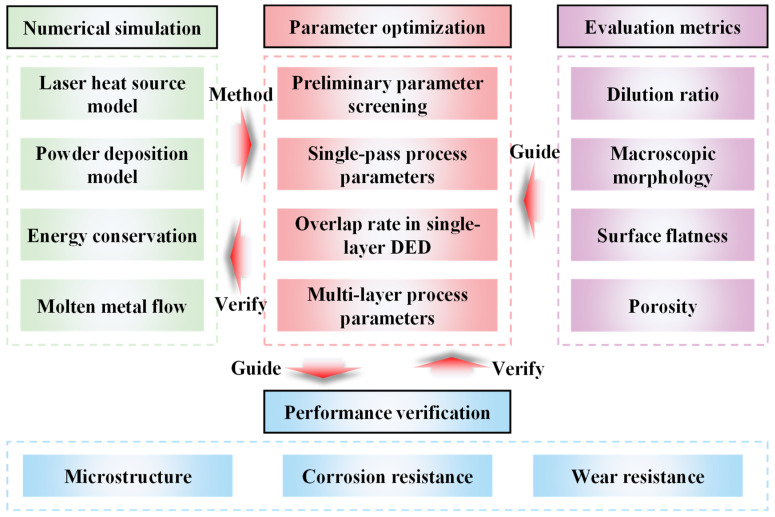
Schematic of the research framework in this work. From the perspective of microstructure, this study integrates numerical simulation with orthogonal experiments to optimize process parameters, followed by comprehensive evaluation of the macroscopic properties of the optimized cladding layers.

In summary, addressing the current research gaps on DED of Hastelloy X on 50CrVA substrates and the limitations of conventional process parameter optimization methods, this study aims to achieve the following objectives: First, to establish a thermo-fluid coupled numerical simulation model for the DED process of Hastelloy X on 50CrVA substrates and validate its accuracy. Second, to develop an integrated optimization method by combining the thermo-fluid coupled model with orthogonal experiments. Third, to assess the corrosion and wear resistance of claddings fabricated by the optimized parameters, verifying whether the Hastelloy X claddings meet the high-performance repair requirements of 50CrVA aircraft landing gear. This work can provide engineering application support for the additive repair of dissimilar 50CrVA landing gears. The specific research framework is illustrated in Figure 1.

## 2. FE Modeling for the DED Process of Hastelloy X on 50CrVA Substrates

**Figure 2:**
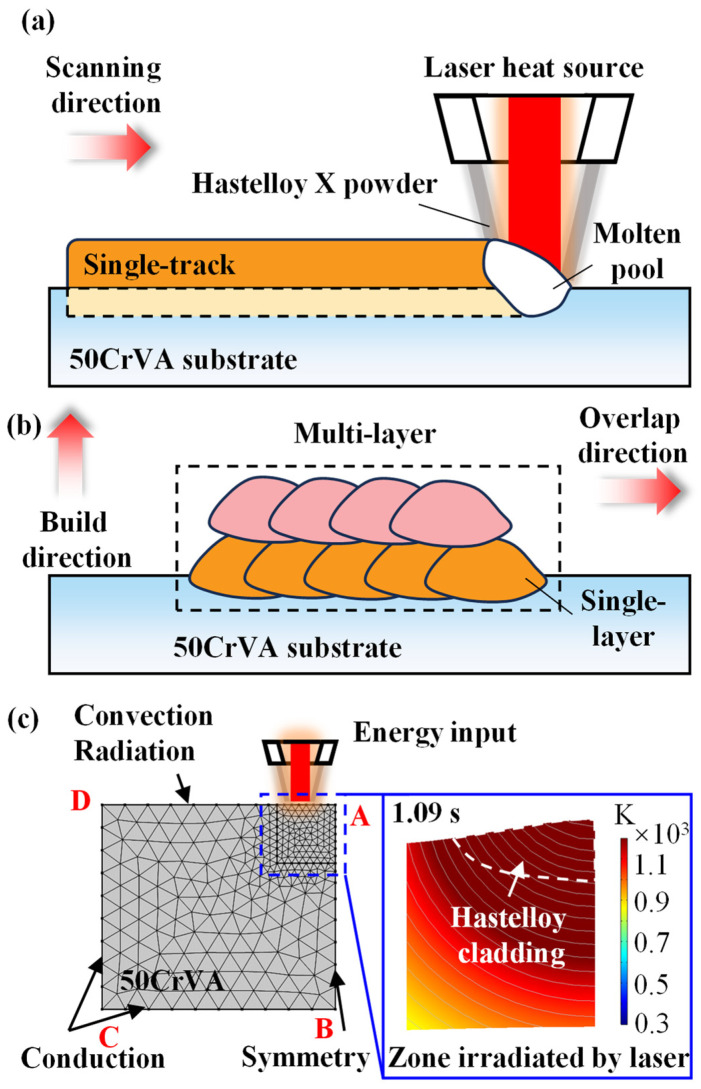
Schematic of the DED principle for multi-layer cladding and the associated simulation model. (**a**) Single-track cladding process; (**b**) Multi-layer cladding process; (**c**) Simulation model. Under laser irradiation, the powder bonds with the substrate surface to form a single-track cladding. Individual tracks are overlapped horizontally at a specific overlap rate to form a uniform single-layer cladding. These single layers are then systematically stacked along the building direction, ultimately forming a multi-layer structure. The model incorporates heat conduction along the BC and CD boundaries and combined heat transfer (conduction, convection, and radiation) on the AD surface.

Figure 2 illustrates the detailed working principle of the DED technology. During the deposition of Hastelloy X, the energy emitted by the laser heat source acts on the substrate surface and the coaxially delivered Hastelloy X powder carried by inert gas, causing the powder to melt and rapidly form a molten pool containing mixed metallic materials at the laser spot. Due to the temperature gradient on the molten pool surface, corresponding surface tension gradients are generated, driving convection within the liquid metal and thereby promoting the redistribution of energy inside the pool. As the laser beam moves along the scanning direction, a new molten pool forms in the directly irradiated zone, while the non-irradiated zone cools and solidifies rapidly, ultimately producing a single-track cladding along the laser path. In Figure 2c, mesh refinement is applied only in the directly laser-irradiated zone to optimize computational efficiency. Boundaries BC and CD are subjected to heat conduction. The AD surface, which is the substrate surface under direct laser irradiation, experiences heat conduction, convection, and radiation due to laser heating. To simplify the computation, a symmetric boundary condition is applied to the AB surface. Since the DED process occurs in a sealed inert gas chamber where gas flow is relatively stable, the convective heat transfer coefficient is set as a constant. The single-track cladding serves as the fundamental building unit. Through horizontal overlapping of individual tracks, a single-layer cladding is formed. These single layers are then stacked vertically, ultimately constructing a multi-layer cladding structure.

The energy distribution of the laser heat source (*I*) can be approximated as a Gaussian distribution. The dynamic movement of the laser beam along the scanning direction can be simulated using a Gaussian heat source moving at the scanning speed. The Gaussian laser heat source can be expressed as [26]:

(1) $$I(x,t)=\frac{2p\eta_{l}}{\pi r^{2}}\exp(-2\frac{{\left(r-vt\right)}^{2}+x^{2}}{r^{2}})-h(T-T_{0})-\sigma_{b}\varepsilon (T^{4}-T_{0}^{4})$$

where *p* represents the laser power, *η_l_* denotes the laser energy absorption coefficient, *r* is the beam radius, *v* is the scanning speed, *x* indicates the distance from the center of the laser beam, *h* is the convective heat transfer coefficient, *T* denotes the processing temperature, *T*_0_ is the ambient temperature, *σ_b_* refers to the Stefan–Boltzmann constant, *ε* is the material absorptivity.

The processes of powder deposition and cladding formation are simulated using a free-surface deformation growth model. The growth velocity (*V_p_*) of the substrate surface is determined by the feed rate of Hastelloy X powder delivered through the coaxial nozzle, which is expressed as [27]:

(2) $$V_{p}=\frac{2f\eta_{p}}{\rho_{p}\pi r_{p}^{2}}\exp(-2\frac{{\left(r-vt\right)}^{2}+x^{2}}{r_{p}^{2}})z$$

where *f* represents the powder feed rate, *η_p_* denotes the powder capture efficiency, *ρ_p_* is the powder density, *r_p_* is the powder stream radius, ***z*** indicates the unit vector along the *Z*-axis.

During the deposition process, energy is input in the form of heat. Both the substrate and the powder undergo solid/liquid phase transformation during melting. Therefore, the influence of latent heat of fusion must be considered in the heat transfer equation. The energy conservation equation is expressed as [28]:

(3) $$\frac{\partial (\rho c_{p}T)}{\partial t}+\frac{\partial (\rho u_{i}c_{p}T)}{\partial x_{i}}=\frac{\partial }{\partial x_{i}}(k\frac{\partial T}{\partial x_{i}})-\frac{\partial \Delta H}{\partial t}-\frac{\partial (\rho u_{i}\Delta H)}{\partial x_{i}}$$

where *ρ* represents the density, *c_p_* denotes the specific heat capacity, *u_i_* is the velocity component in the *i*-th direction, *k* is the thermal conductivity coefficient, Δ*H* indicates the latent heat of fusion. The thermophysical properties of the solid–liquid mixed phase can be calculated using a weighted average based on the mass/volume fraction ratio of the solid and liquid phases.

The incompressible Navier–Stokes (N-S) equations are employed to describe the flow of liquid metal within the molten pool. The mass and momentum conservation equations can be expressed as:

(4) $$\frac{\partial \rho }{\partial t}+\frac{\partial (\rho u_{i})}{\partial x_{i}}=0$$

(5) $$\frac{\partial (\rho u_{i})}{\partial t}+\frac{\partial (\rho u_{i}u_{j})}{\partial x_{i}}=\frac{\partial }{\partial x_{i}}(\mu \frac{\partial u_{j}}{\partial x_{i}})-\frac{\partial p}{\partial x_{i}}+\frac{\partial }{\partial x_{j}}(\mu \frac{\partial u_{j}}{\partial x_{i}})-K_{0}\frac{{\left(1-f_{l}\right)}^{2}}{f_{l}^{3}+B}u_{i}+F$$

where *μ* represents the dynamic viscosity, *p* denotes the pressure, *K*_0_ is a constant characterizing the morphology of the porous media, *B* is a small numerical value introduced to prevent division by zero in the viscous dissipation term, *F* indicates the coupled effects of gravity, surface tension of the molten pool, and volumetric forces (N/m^3^), which are expressed as:

(6) F=ρgβ(T−T0)+ρg+γ∇Tt+σκz

where *g* represents the gravitational acceleration (m/s^2^), *β* denotes the thermal expansion coefficient of the metal, *γ* is the temperature coefficient of surface tension, *σ* is the surface tension, *κ* indicates the interface curvature.

**Figure 3:**
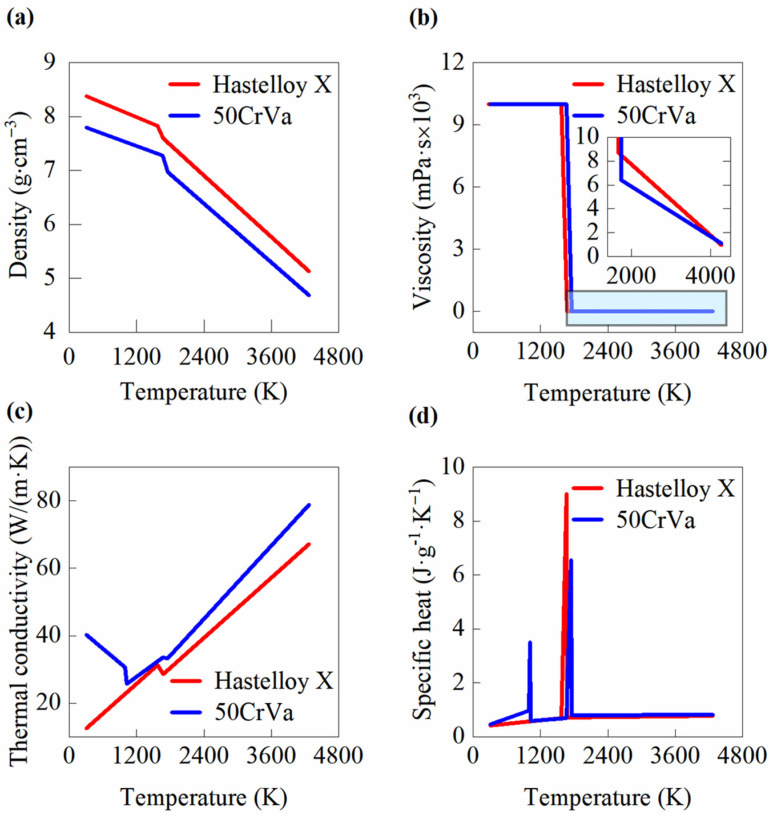
Temperature-dependent thermal properties of 50CrVA and Hastelloy X materials. (**a**) Density; (**b**) Viscosity; (**c**) Thermal conductivity; (**d**) Specific heat. The thermal properties of the material change at the solid–liquid phase transition temperature (as seen in Table 1).

The constant thermal property parameters of the materials during the simulation are listed in Table 1. Other temperature-dependent parameters are derived from thermodynamics and kinetics calculations, with their linearly fitted curves shown in Figure 3. Since the viscosity of the material in the solid state is negligible, the solid-phase viscosity is set to an extremely high value to facilitate subsequent computations. The density and viscosity of both materials decrease with increasing temperature. When the temperature reaches the solid–liquid phase transition point, both density and viscosity undergo abrupt changes. The thermal conductivity of Hastelloy X generally exhibits a positive correlation with temperature. However, during the solid–liquid phase transition, the thermal conductivity slightly decreases as temperature increases. In contrast, the thermal conductivity of 50CrVA initially decreases but increases with rising temperature when exceeding 1100 K. Similarly, within the temperature range of the solid–liquid phase transition, the thermal conductivity shows a slight decrease as temperature rises. The specific heat curves of Hastelloy X and 50CrVA generally exhibit similar trends. As temperature increases, the specific heat capacity gradually rises until stabilizing. During the solid–liquid phase transition, the specific heat first increases sharply and then decreases rapidly. For 50CrVA, it is particularly noteworthy that the specific heat undergoes a significant change around 1100 K, following a trend similar to that observed during the phase transition. The parameters of the mixed molten metal pool can be calculated using the rule of mixtures formula.

## 3. Experimental Details

### 3.1. Materials and DED Equipment

The substrate material used in this study was 50CrVA spring steel (Liaoning Guanda New Material Technology Co., Ltd., Anshan, China), a commonly used material for critical aircraft landing gear components, with dimensions of 100 mm × 100 mm × 20 mm. The powder material was Hastelloy X (Liaoning Guanda New Material Technology Co., Ltd., Anshan, China), with a particle size range of 40~90 μm. Its chemical composition is provided in Table 2. Prior to the experiment, the Hastelloy X powder was dried in a vacuum oven at 373.15 K for 2 h. The oxide layer on the substrate surface was removed by milling, followed by cleaning with alcohol to prevent reoxidation.

**Figure 4:**
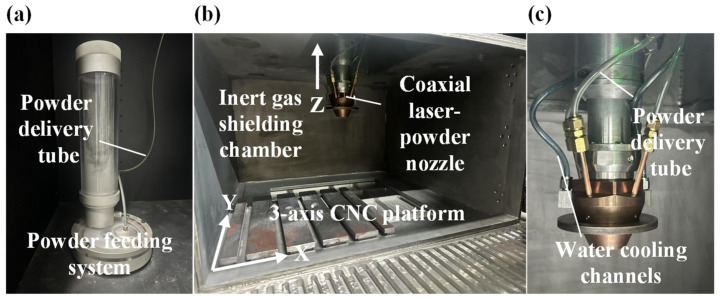
The self-developed DED equipment. (**a**) Powder feeding system; (**b**) Inert gas shielding chamber with a 3-axis CNC platform; (**c**) Coaxial laser–powder nozzle.

The experimental setup consisted of a self-developed DED additive manufacturing system, primarily including an inert gas-protection chamber, a powder feeding system (Nanjing Zhongke Raycham Laser Technology Co., Ltd., Nanjing, China), a three-axis CNC platform, and a coaxial laser–powder nozzle (RFL-A2000D, Wuhan Raycus Fiber Laser Technologies Co., Ltd., Wuhan, China). The equipment is illustrated in Figure 4. During the experiments, the powder feeding system operated with an argon flow rate ranging from 8.9 L/min to 9.0 L/min to purge the environment, while the Hastelloy X powder was delivered through the powder feeding tube via an argon carrier gas. To prevent powder contamination of the laser optical path, the shielding gas flow rate was maintained at 8 L/min. The substrate was fixed on the three-axis CNC platform and moved accordingly.

To characterize the morphological parameters (height *H*, width *W*, and depth *D*) of the single-track cladding prepared by DED, samples were prepared for metallographic analysis. Stable cross-sections of the cladding layers were obtained using wire cutting and a metallographic sample cutter. The cross-sections were ground with abrasive papers and subsequently polished with diamond suspension. The polished samples were cleaned with alcohol and dried with compressed air. The samples were observed under an inverted metallurgical microscope (Ningbo Sunny Instruments Co., Ltd., Yuyao, China) to record the morphological parameters of the cladding layers.

### 3.2. Method of Process Parameter Optimization

To improve experimental efficiency, the finite element method (FEM) was first utilized to independently optimize the parameters within the specified ranges of laser power (*p*, 1500~2200 W), powder feed rate (*f*, 1.0–4.5 g/min), and scanning speed (*v*, 100~450 mm/min). Based on the preliminary optimization, orthogonal experiments were conducted within the refined ranges of *p*, *f*, and *v* to systematically determine the optimal parameter combinations. The dilution ratio (*η*) of the cladding layer represented the ratio of the melted depth to the total height. The calculation formula for the dilution ratio is shown in Equation (7). A higher *η* indicated that there was excessive laser energy acting on the substrate, leading to overwhelming melting and potential collapse. A low *η* suggested insufficient laser–substrate interaction, resulting in minimal melting and poor metallurgical bonding. Therefore, the process parameters were optimized targeting an *η* value between 35% and 60% as the evaluation criterion.

(7) $$\eta =\frac{D}{D+H}\times 100\%$$

Within the parameter range obtained from simulations, single-track DED experiments were conducted by varying *p*, *f*, and *v*. Based on the macroscopic morphology of the cladding layers, including surface ablation marks and finish quality, a suitable parameter range for depositing high-quality single-track Hastelloy X on 50CrVA substrates was determined. Subsequently, the influence of overlap ratio on the surface quality of single-layer cladding was experimentally investigated. The optimal overlap ratio was determined by optimizing the surface flatness (*ζ*) of the single layer. The overlap rate is defined as the percentage of the overlapping width between two adjacent single tracks relative to the width of a single track. Within the preferred single-track parameter range, six overlap rate levels (35%, 40%, 45%, 50%, 55%, 60%) were selected for single-layer deposition experiments. The *ζ* of each sample was quantitatively analyzed by evaluating the ratio of the minimum to maximum layer height within the effective central region, excluding the non-overlapping semicircular portions of the tracks. As *ζ* approaches 1, the maximum and minimum layer heights converge, indicating increasingly uniform cladding surface. Finally, orthogonal experimental design was employed to minimize the porosity of multi-layer claddings, using porosity as the evaluation metric. Here, porosity refers to the ratio of the total area of all pores on the cross-section of the multi-layer claddings to the area of that cross-section. The original images of the cladding cross-sections were binarized, with black pixels representing pores and white pixels representing defect-free zones. The porosity of the multi-layer cladding cross-section was calculated as the percentage of black pixels relative to the total number of black and white pixels in the binarized image. To more accurately determine the porosity on the metallographic sample surface, eight randomly selected regions of 3000 × 2000 pixels were analyzed on the cross-section of the multi-layer cladding. The porosity in each zone was calculated, and the average value was taken. Three factors (*p*, *f*, *v*) were tested at three levels each, requiring a total of nine experimental groups. The parameter combination with the lowest porosity was identified, and the results of the orthogonal experiments were evaluated using three-factor analysis of variance (ANOVA) and range analysis.

### 3.3. Corrosion and Wear Performance Verification

The polished samples were chemically etched for 30 min using a solution composed of 50 mL H_2_O:15 mL HCl:6 g FeCl_3_. After etching, surface contaminants were removed with anhydrous ethanol to facilitate observation of the metallographic microstructure. Corrosion measurements were conducted using an electrochemical workstation with a three-electrode cell, in which a 3.5 wt% NaCl solution served as the electrolyte. The samples (optimized cladding, pre-optimization cladding, and substrate) were ground, polished, and cold-mounted, exposing only a 1 cm^2^ test surface to prevent extraneous corrosion effects. The open circuit potential (OCP) was first measured. Polarization curves were then scanned within OCP ± 100 mV at a rate of 3 mV/s with a step interval of 5 mV. Subsequently, electrochemical impedance spectroscopy was performed with an AC amplitude perturbation of 5 mV over a frequency range of 5 × 10^−2^ Hz to 1 × 10^6^ Hz. The hardness of the cladding layers was measured using a Cratos W50S Vickers hardness tester under a load of 500 g and a dwell time of 10 s. The wear resistance of the samples was evaluated with a multi-functional tribometer. All samples were ground and polished to ensure a scratch-free surface. The test parameters are listed in Table 3. The friction coefficient was determined in real time from the normal force and friction force values recorded by the sensor (one test per sample). To further assess wear resistance, the groove width generated during testing was examined using an inverted metallurgical microscope. For each sample, the groove width was measured three times at uniform locations under identical test conditions, with wider grooves indicating inferior wear resistance of the material.

## 4. Results and Discussion

### 4.1. Determination of Process Parameters for High-Quality Single-Track Cladding Based on a Thermal–Flow Coupled Model and Single-Factor Experiments

**Figure 5:**
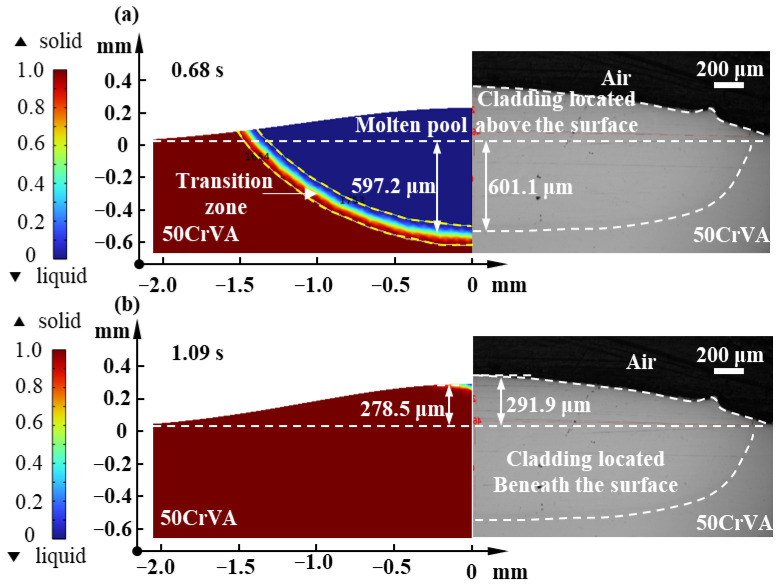
Comparison between simulated and experimental results: (**a**) Depth; (**b**) Height. The relative errors of height and depth are 4.6% and 0.6%, respectively. The prediction accuracy of the FEM is high.

To establish an optimization method of the process parameters for single-track cladding, this section first validates the thermal–flow coupled FEM. Under 2000 W in *p*, 1.5 g/min in *f*, and 210 mm/min in *v*, the calculated and experimentally measured molten pool dimensions (*D* and *H*) are compared. As shown in Figure 5, the simulated cladding exhibits 278.5 μm in *H* and 597.2 μm in *D*, while the experimental results show 291.9 μm in *H* of and 601.1 μm in *D*. In this simulation, the laser heat source continuously inputs energy into the substrate surface over time, leading to a rise in substrate temperature. Surface melting occurs, and the molten Hastelloy forms a molten pool. When the time reaches 0.68 s, the molten pool reaches its maximum depth, which is defined as the cladding depth. At this stage, the molten pool has not yet cooled, and additional Hastelloy powder continues to enter the molten pool, meaning the molten height at this stage does not represent the final cladding height. When the time reaches 1.09 s, the molten pool has almost completely cooled, and the molten height at this moment is considered the final cladding height. The relative errors between the simulated and experimental results are calculated to be 4.6% in height and 0.6% in depth, indicating good agreement and validating the effectiveness of the model for subsequent optimization experiments of DED parameters.

**Figure 6:**
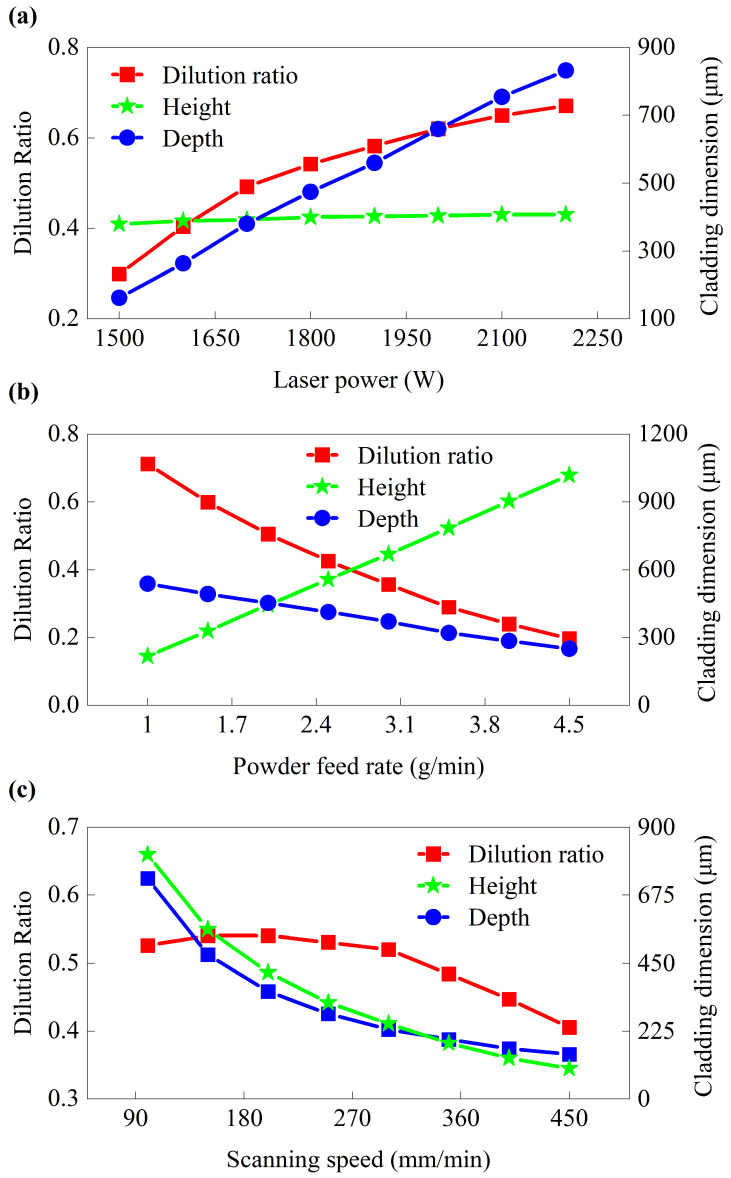
Dilution rate, height, and depth of the cladding layer with the change of process parameters using the FEM. (**a**) *f* = 1.8 g/min and *v* = 180 mm/min; (**b**) *p* = 1800 W and *v* = 180 mm/min; (**c**) *p* = 1800 W and *f* = 1.8 g/min.

Using the validated FEM, single-factor experiments are conducted to determine the preferred range of DED process parameters, with results presented in Figure 6. As illustrated in Figure 6a, under constant *f* and *v*, *D* and *η* increase rapidly with rising *p*, exhibiting a steep trend in depth, while the change in *H* is relatively small. This reason is that increased *p* delivers more energy to the substrate, forming a larger molten pool and thus deepening the cladding. At a fixed *f*, higher *p* melts more powder into the pool, leading to a minor increase in *H*. However, the rate of increase in *H* is significantly lower than that in *D*, resulting in an elevated *η*. Figure 6b demonstrates that, at constant *p*, increasing *f* diverts more energy toward melting the powder rather than the substrate, thereby reducing *D*. Meanwhile, the increased powder input substantially raises *H*, causing *η* to decrease with higher *f*. In Figure 6c, *η* initially rises and then declines as *v* increases. Higher *v* reduces energy input and powder deposition per unit length, decreasing both *D* and *H* of the cladding. At lower *v*, *H* decreases more rapidly, leading to an increase in *η*. Beyond 300 mm/min, the reduction in *D* becomes dominant, reversing the trend. Specifically, when *v* increases from 200 mm/min to 350 mm/min, *D* and *H* decrease by 67.1% and 58.8%, respectively, while *η* experiences a slight increase. Above 300 mm/min, the cladding depth collapses to 100~200 μm, indicating poor bonding with the substrate. To ensure substrate adhesion and prevent cladding collapse in subsequent experiments, *η* of 35%~60% is adopted as the optimization criterion. Therefore, the preliminary ranges in *p*, *f*, and *v* are 1600~2000 W, 1.2~3.0 g/min, and 100~300 mm/min, respectively.

**Figure 7:**
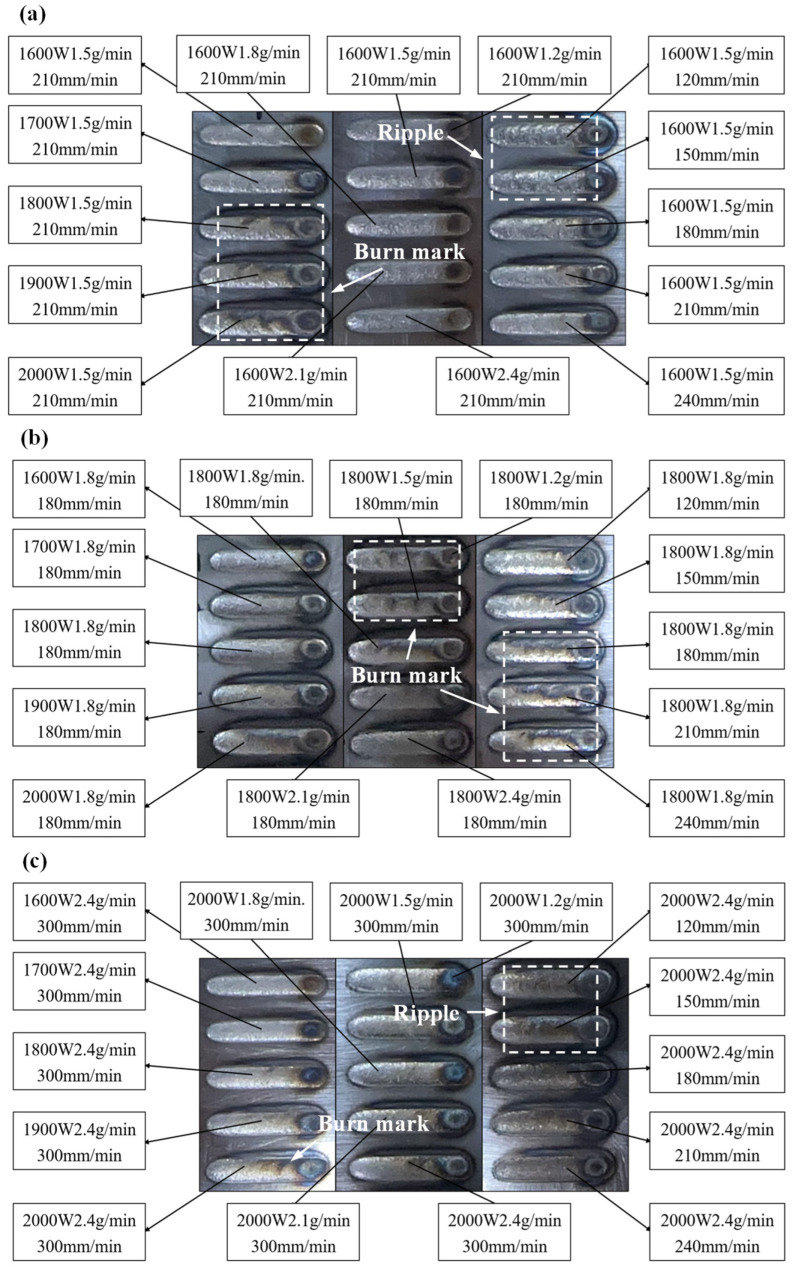
Macroscopic morphology of the cladding prepared in the three experimental groups: (**a**) Single-factor experiments based on 1600 W, 1.5 g/min, 210 mm/min; (**b**) Single-factor experiments based on 1800 W, 1.8 g/min, 180 mm/min, (**c**) Single-factor experiments based on 2000 W, 2.4 g/min, 300 mm/min.

Based on the process parameter range determined by FEM, three sets of DED experiments are conducted within refined ranges of 1600~2000 W in *p*, 1.2~2.4 g/min in *f*, and 120~240 mm/min in *v*, as presented in Figure 7. In Figure 7a, when *p* reaches 1800~2000 W, excessive energy input causes visible ablation marks on the cladding surface. At *f* of 1.2~2.4 g/min, the surface remains relatively smooth with a metallic luster. However, at lower *v* (120~150 mm/min), intense fluid flow within the molten pool due to high energy input per unit length results in periodic surface ripples. In Figure 7b, ablation marks appear at lower *f* (1.2~1.8 g/min) combined with higher *p*. Sustained burning at *v* of 180~240 mm/min is attributed to insufficient cooling of the substrate between depositions. During sequential cladding, the initial temperature of the substrate gradually increases from room temperature, requiring less energy, to reach melting point in subsequent tracks under the same parameters. To avoid this, a 1 min cooling interval between depositions is implemented, as depicted in Figure 7c. At *p* of 1600~2000 W, the surface is smooth, but periodic ripples persist at *v* of 120~150 mm/min. To sum up, ablation marks occur at high *p* or low *f*, while low *v* induces ripples through unstable molten pool dynamics. Inter-track cooling effectively mitigates cumulative heating effects. Through a combined approach of simulation and experiments, the optimal process parameter range was determined by comprehensive evaluation of *η* and surface morphology. The results demonstrate that single-track cladding with superior surface quality is achieved within the ranges of 1600~1800 W in *p*, 1.8~2.4 g/min in *f*, and 150~240 mm/min in *v*. These process parameters can serve as a reference for subsequent fabrication of high-quality Hastelloy X cladding layers.

### 4.2. Optimization of Process Parameters for Multi-Layer Cladding by Considering Surface Flatness and Porosity

**Figure 8:**
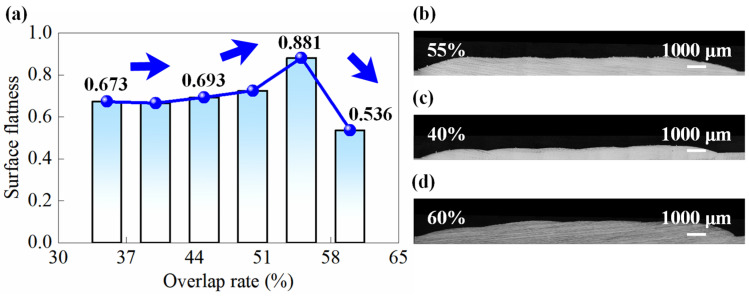
Experiment results of overlap rate optimization. (**a**) Variation of surface flatness with increasing overlap rate for single-layer cladding; (**b**–**d**) are cross-sections of single-layer cladding at the overlap rates of 55%, 40%, and 60%, respectively. As the overlap rate increases, the surface flatness of the single-layer cladding first improves and then deteriorates, reaching an optimal value of 0.88 at an overlap rate of 55%.

To obtain the optimal DED parameters for multi-layer structures, this section first focuses on optimizing the overlap rate during single-layer cladding. Within the determined range of single-track parameters, a parameter combination of 1600 W in *p*, 1.5 g/min in *f*, and 180 mm/min in *v* is selected to conduct single-layer cladding experiments at different overlap rates. The variation of *ζ* with increasing overlap rate is presented in Figure 8. The *t* initially increases and then decreases with increasing overlap rate, which is consistent with the trend observed in [29]. The reason is that the inherent morphology of single tracks exhibits an axisymmetric convex shape, with the center higher than the edges. When adjacent tracks overlap horizontally, vertical stacking occurs in the overlapping zone. Additionally, during the deposition of subsequent tracks, partial remelting occurs in the overlapping zone of the previous track, leading to increased *D* in this region. Under the combined effect of these mechanisms, the *H* in the overlap zone becomes slightly greater than the original track height. When the overlap rate is below 55%, increasing the overlap rate brings the height (H) of the overlapping zone closer to the original track height, thereby improving *ζ*. However, at an overlap rate of 60%, the *H* in the overlapping zone exceeds the original track height. This results in an inclined surface where the start side of deposition is lower than the end side, significantly reducing *ζ*. Based on these observations, an overlap rate of 55% is selected for subsequent single-layer cladding experiments to achieve the optimal surface quality.

**Figure 9:**
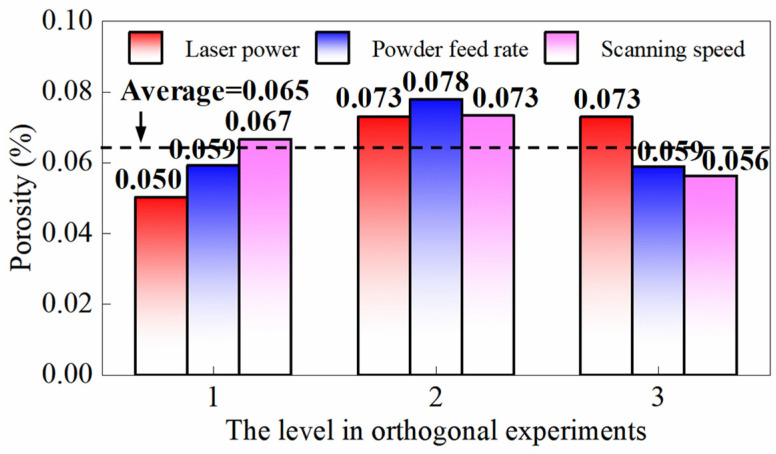
The porosity of multi-layer cladding at different levels for the three factors indicates laser power and powder feed rate exert significant influences on porosity. The minimum porosity is achieved when the laser power is set to level 1 and the powder feed rate is set to level 3.

Orthogonal experiments are conducted with the porosity of multi-layer cladding as the optimization objective. The factors and their corresponding levels in the orthogonal experiment are listed in Table 4. The porosities of multi-layer claddings generated under the nine parameter sets are summarized in Table 5. The results of the three-factor ANOVA are presented in Table 6, and the range analysis results are depicted in Table 7. The range analysis calculates the average porosity values for each factor at its three levels based on the data in Table 5, reflecting the relative influence of each factor on porosity. The variation trends of porosity in multi-layer claddings across different factor levels are illustrated in Figure 9, which presents the range analysis results categorized by different levels to facilitate the analysis of influence of each factor on porosity. As shown in Table 6, at a significance level of α = 0.05, *p* (F = 21.301, P = 0.045 < 0.05) and *f* (F = 19.113, P = 0.050 ≤ 0.05) exhibit significant effects on the porosity in the cladding, while *v* shows no significant influence. The range analysis results show the impact of each factor on the target response. According to Table 7, *p* has the greatest effect on the porosity, followed by *f*, while *v* has the least impact, which is consistent with the three-factor ANOVA results. In Figure 9, within a small range of higher laser power, porosity is positively correlated with *p*. Increased *p* enhances energy input to the molten pool, intensifying fluid flow. This stronger turbulence tends to entrap the argon shielding gas within the molten pool, resulting in pore formation. According to the results of the orthogonal experiment, the process parameter set for the minimum porosity should be (1600 W, 2.4 g/min, 240 mm/min).

**Figure 10:**
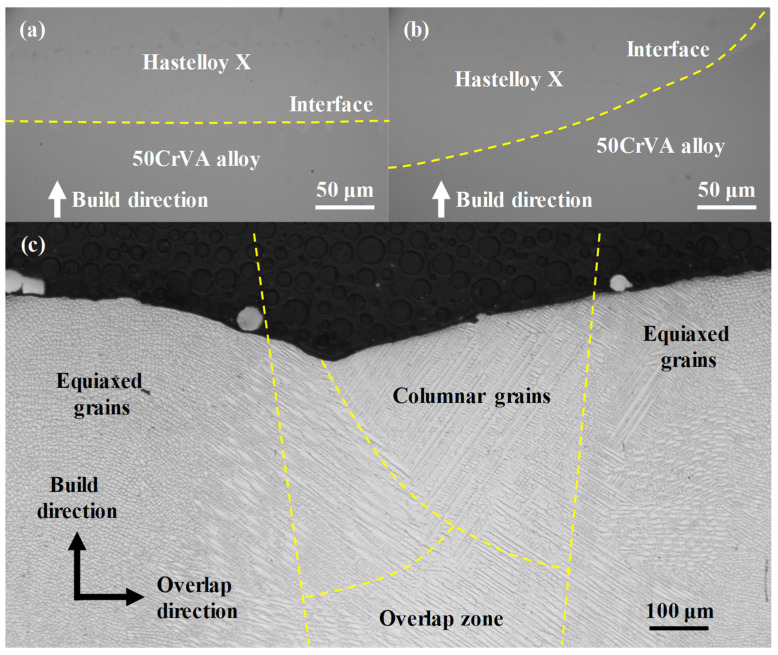
Cladding–substrate interface and microstructure of the cladding layer: (**a**) Bottom interface between the cladding layer and substrate; (**b**) Side interface between the cladding layer and substrate; (**c**) Microstructure near the overlapping zone.

To observe the metallurgical bonding between the cladding layer and the substrate, the cladding layer is etched and examined for its microstructure. The microstructure of the cladding and its interface with the substrate is shown in Figure 10. The interface between the Hastelloy X cladding and the 50CrVA substrate is free of visible cracks or pores in Figure 10a,b, indicating excellent metallurgical bonding. Figure 10c reveals that the cladding contains no significant defects and consists mainly of columnar and equiaxed grains. The equiaxed grains are primarily distributed in the central regions of a single track, while the columnar grains are mainly located in the overlapping zones between adjacent tracks. During deposition, the extremely high cooling rate following the laser beam passage promotes grain nucleation over grain growth, resulting in the formation of fine equiaxed grains. Meanwhile, since the growth direction of grains follows the inverse thermal gradient, the columnar grains in the overlap zones grow perpendicular to the molten pool boundaries. This distinctive grain structure distribution is characteristic of the DED process under high thermal gradients and rapid cooling conditions [30]. In summary, an overlap rate of 55% ensures optimal *ζ* in single-layer cladding. By selecting the parameter combination of *p* at level 1 (1600 W), *f* at level 3 (2.4 g/min), and *v* at level 3 (240 mm/min), multi-layer cladding with minimal porosity can be achieved. Using these optimized parameters, a high-quality Hastelloy X layer can be fabricated on the 50CrVA substrate, characterized by a dense microstructure and excellent interfacial bonding.

### 4.3. Performance Verification of Corrosion and Wear Resistance for Multi-Layer Cladding Fabricated by Optimized Process Parameters

**Figure 11:**
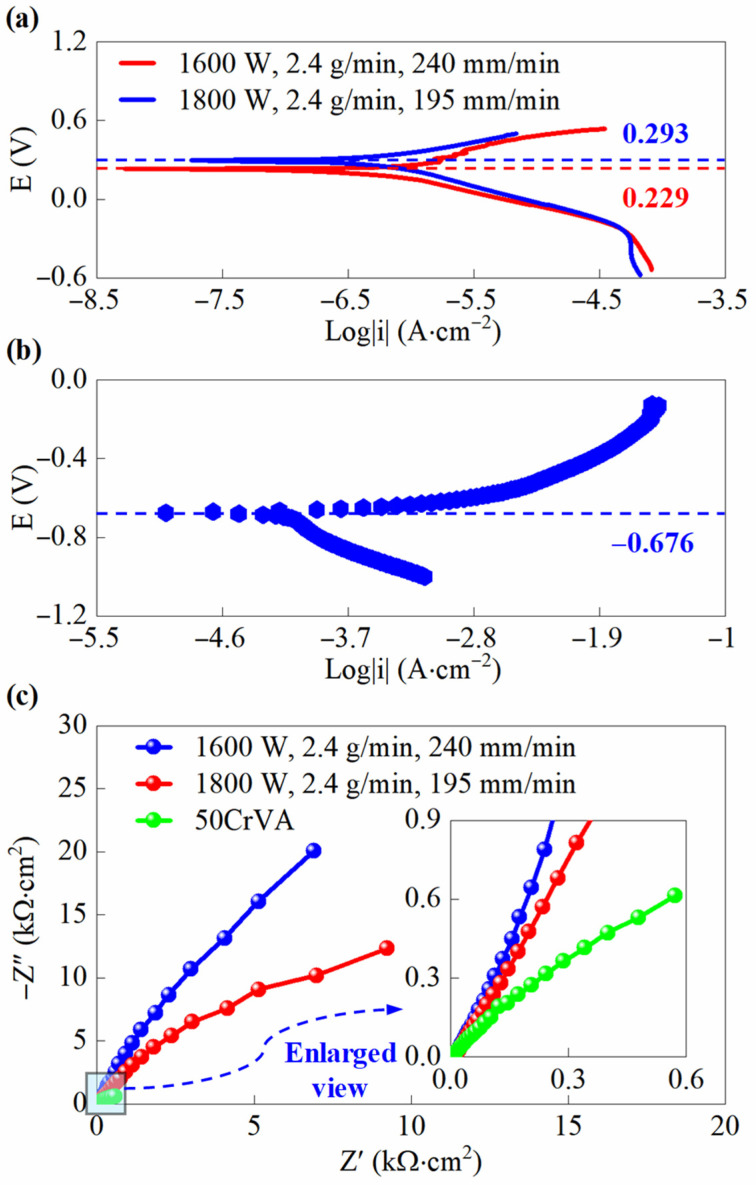
Polarization curve and impedance spectra of the cladding before and after process parameter optimization and substrate, where (1600 W, 2.4 g/min, 240 mm/min) represents the optimized parameter set and (1800 W, 2.4 g/min, 195 mm/min) corresponds to the pre-optimization parameters: (**a**) The polarization curve of cladding; (**b**) The polarization curve of substrate; (**c**) The impedance spectra of the cladding and the substrate.

To verify the reliability of the proposed optimization method, this section first tests the corrosion resistance of cladding layers deposited using both pre-optimized and optimized process parameters, as well as that of the substrate. The polarization curves and impedance spectra of the cladding samples and the substrate are depicted in Figure 11. As seen in Figure 11a, the polarization curves of the cladding layers before and after optimization exhibit similar trends. The corrosion potential of the optimized cladding is 0.229 V, while that of the pre-optimization cladding is 0.293 V, indicating that the voltage required to initiate corrosion is nearly the same in both cases. However, the optimized cladding curve shows a distinct leftward shift, indicating lower current during corrosion, which suggests a reduced corrosion rate. Figure 11b reveals that the substrate has a corrosion potential of −0.676 V, which is substantially lower than that of the cladding. Moreover, the substrate shows much higher current during corrosion compared to the cladding. The corrosion current of the substrate ranges approximately from 10^−4.6^ to 10^−1.9^ A/cm^2^, while that of the cladding layer falls within the range of 10^−7.5^ to 10^−4.5^ A/cm^2^.

The corrosion current of the cladding layer is significantly lower than that of the substrate, approximately 0.001 times of the latter. This demonstrates that the substrate is more prone to corrosion and undergoes more severe corrosion damage. This difference can be attributed to the fact that the Hastelloy X cladding contains abundant Cr, Mo, and other elements that readily form a dense passive film [31,32], preventing corrosive media from penetrating into the material and thereby reducing the corrosion rate. The substrate has lower Cr content and contains Fe, making it more susceptible to corrosion with poorer corrosion resistance. The optimized cladding contains fewer pores and has a more compact microstructure, resulting in an even denser passive film that provides enhanced corrosion inhibition. Figure 11c shows that the impedance spectra of all samples exhibit semicircular arcs, where a larger arc radius indicates better corrosion resistance [32]. The optimized cladding demonstrates a larger radius compared to the pre-optimized cladding, revealing that porosity significantly affects the impedance characteristics of the cladding. Specifically, lower porosity corresponds to improved corrosion resistance of the cladding. In contrast, the substrate exhibits a much smaller impedance arc radius than the cladding, confirming its inferior corrosion resistance. This observation aligns well with the polarization curve analysis, further verifying that the claddings offer superior corrosion protection compared to the substrate.

**Figure 12:**
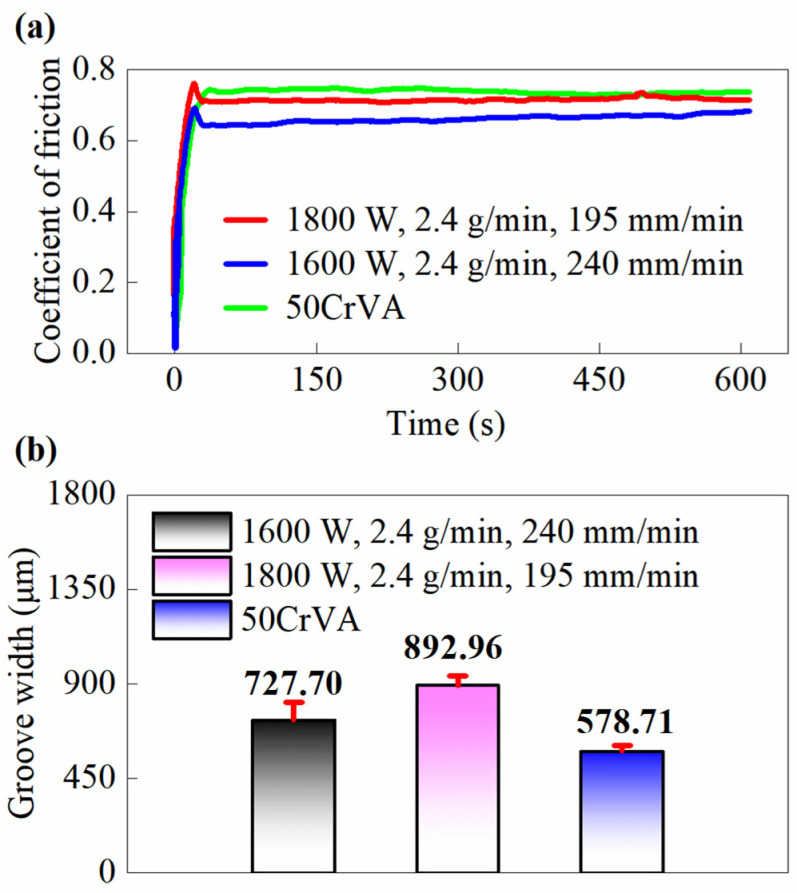
Friction coefficient and groove width of the cladding layer (before and after process parameter optimization) and substrate, where (1600 W, 2.4 g/min, 240 mm/min) represents the optimized parameter set and (1800 W, 2.4 g/min, 195 mm/min) corresponds to the pre-optimization parameters: (**a**) Coefficient of friction; (**b**) Groove width.

To further evaluate the wear resistance of the cladding layer, hardness and ball-on-disk wear tests are conducted. The hardness of the cladding (before and after optimization) and the substrate is presented in Table 8. Figure 12 shows the variation of friction coefficient over time and the wear conditions of different samples. As can be seen from Figure 12a, during the initial stage (0~10 s), the gas or contaminant films (such as oxide layers and water vapor) adsorbed on the material surface are destroyed during the initial sliding, exposing fresh metal surfaces. This leads to an increased direct contact zone and a significantly enhanced adhesive friction component. Meanwhile, the interlocking of microscopic asperities between the sample and grinding ball strengthens mechanical interlocking, requiring greater shear force to overcome and resulting in a rapid upward trend of friction coefficient. After a certain period (10~15 s), continuous sliding of the grinding ball causes fracture and pulverization of surface asperities, reducing surface roughness of both contact surfaces and weakening mechanical interlocking. Local temperature rise during friction reduces the material hardness and decreases plowing resistance. However, as thermal equilibrium has not yet been reached at this stage, the coefficient of friction shows a slight decrease. When stable friction is achieved (>15 s), the friction coefficients of cladding before and after optimization show little difference and are slightly higher than that of the substrate. Figure 12b shows that the wear mark of the optimized cladding is narrower than that of the pre-optimization cladding, indicating that porosity affects the wear resistance of cladding. The wear marks of the cladding are slightly wider than that of the substrate, demonstrating that the substrate has better wear resistance than the cladding. The reason is that the pores in the cladding increase resistance during sliding, and lower porosity results in better wear resistance. Generally, the wear resistance of materials should be positively correlated with their hardness [33]. As shown in Table 8, the substrate has the highest hardness, while the hardness values of the optimized and pre-optimized cladding are similar. Therefore, the substrate exhibits better wear resistance than the cladding layer.

## 5. Conclusions

The DED technology using Hastelloy X offers a highly promising method for repairing surface defects in heterogeneous 50CrVA landing gear components of aircraft. In this work, an optimization method combining the thermal–flow coupled model with orthogonal experiments is proposed. Meanwhile, the preferred process parameters for each stage of multi-layer cladding are determined from the perspective of dilution ratio, microscopic morphology, surface flatness, and porosity. Moreover, the corrosion and wear resistance of the repaired workpiece are comprehensively evaluated, verifying the preparation of high-quality Hastelloy X claddings on 50CrVA substrates via the DED process. The main conclusions can be drawn as follows:

1. A thermal–flow coupled finite element model for the DED process of Hastelloy X on 50CrVA substrates is built and its model accuracy is less than 5%. Combined with experimental results, the preferred range of process parameters for single-track cladding is determined to be 1600~1800 W in laser power, 1.8~2.4 g/min in powder feed rate, and 150~240 mm/min in scanning speed.

2. The influence of overlap rate on the surface flatness (*ζ)* of single-layer cladding is investigated. Local height variations caused by remelting and material accumulation in the overlap zone led to a non-monotonic trend where ζ first increased and then decreased with increasing overlap rate. At an overlap rate of 55%, an optimal *ζ* of 0.88 is achieved, corresponding to the most uniform height distribution on the cladding surface.

3. Orthogonal experimental design is employed with porosity as the optimization criterion for selecting process parameters in multi-layer cladding. Three-factor analysis of variance and range analysis reveal that laser power (*p)* and powder feed rate (*f)* are the dominant factors influencing porosity. Within a small range of higher laser power, porosity shows a positive correlation with *p*.

4. An overlap rate of 55% and an optimized process parameter set (1600 W, 2.4 g/min, 240 mm/min) achieve the lowest internal porosity of multi-layer cladding. Under these conditions, the cladding structure exhibits excellent interface quality between Hastelloy X and the 50CrVA substrate, along with a dense microstructure free of detectable cracks or pores.

5. The corrosion and wear resistance of the high-quality multi-layer cladding are evaluated. The optimized cladding exhibits a corrosion potential comparable to that of the pre-optimized cladding, with a lower corrosion current density and higher electrochemical impedance. The corrosion resistance of the cladding layer is significantly higher than that of the substrate. The friction coefficient remains unchanged and wear resistance is significantly enhanced for the optimized cladding.

This study provides theoretical guidance for repairing components made of dissimilar powder materials using directed energy deposition technology, offering substantial practical application value.

## Figures and Tables

**Table 1:** Thermal property parameters of the 50CrVA substrate material and Hastelloy X powder.

Thermal Properties	Unit	Value
Solidus temperature of the powder	K	1568.9
Liquidus temperature of the powder	K	1670.4
Phase transformation temperature of the powder	K	1619.7
Transformation interval of the powder	K	66.3
Latent heat of the powder	J/g	299.7
Solidus temperature of the substrate	K	1663.3
Liquidus temperature of the substrate	K	1752.6
Phase transformation temperature of the substrate	K	1707.9
Transformation interval of the substrate	K	89.3
Latent heat of the substrate	J/g	308.8

**Table 2:** The mass fraction of each chemical element in Hastelloy X (wt%).

Cr	Fe	Co	Mo	Al	W	Ti	Si	Mn	Cu	C	Ni
22.33	19.07	1.33	8.72	0.014	0.57	0.01	0.03	0.0054	0.005	0.069	Bal.

**Table 3:** Test parameters for wear performance evaluation of Hastelloy X and 50CrVA materials.

Parameters	Temperature (K)	Load (N)	Rotational Speed (rpm)	Time (min)	Ball Diameter (mm)	Ball Material
Value	293.15	5	100	10	2.38	Si_3_N_4_

**Table 4:** Factors (laser power, powder feed rate, and scanning speed) and level settings in the orthogonal experiment.

Factors	Level		
	1	2	3
Laser power (W)	1600	1700	1800
Powder feed rate (g/min)	1.8	2.1	2.4
Scanning speed (mm/min)	150	195	240

**Table 5:** Porosity of multi-layer cladding obtained by orthogonal experiments under various process parameters.

No.	Factors			Porosity (%)	
	Laser Power (W)	Powder Feed Rate (g/min)	Scanning Speed (mm/min)	Average	Standard Deviation
1	1600	1.8	150	0.054	0.023
2	1600	2.1	195	0.066	0.067
3	1600	2.4	240	0.031	0.041
4	1700	1.8	195	0.071	0.059
5	1700	2.1	240	0.085	0.135
6	1700	2.4	150	0.063	0.065
7	1800	1.8	240	0.053	0.044
8	1800	2.1	150	0.083	0.122
9	1800	2.4	195	0.083	0.161

**Table 6:** ANOVA results of the three factors (laser power, powder feed rate, and scanning speed).

Scheme.	Sum of Squares	Degrees of Freedom	Mean Square	F	P
Laser power	0.001022742	2	0.000511371	21.301	0.045
Powder feed rate	0.000718039	2	0.000359020	19.113	0.050
Scanning speed	0.000436842	2	0.000218421	6.267	0.138
Error	0.000323292	2	0.000161646	/	/
Total	0.002500915	8	/	/	/

**Table 7:** Range analysis results of three factors (laser power, powder feed rate, and scanning speed).

Mean Value at Each Level	Laser Power (W)	Powder Feed Rate (g/min)	Scanning Speed (mm/min)
1	0.05037	0.05912	0.06667
2	0.07344	0.07807	0.07329
3	0.07250	0.05912	0.05636
Range	0.02307	0.01895	0.01693
Order	1	2	3

**Table 8:** Hardness of the cladding before and after optimization and substrate.

Samples	Average Hardness (HV0.5)
Pre-optimization cladding	223.21
Optimized cladding	222.33
Substrate	288.87

## Data Availability

Data will be made available on request.
